# Radiation as an immunological adjuvant: current evidence on dose and fractionation

**DOI:** 10.3389/fonc.2012.00153

**Published:** 2012-10-26

**Authors:** Sandra Demaria, Silvia C. Formenti

**Affiliations:** ^1^Department of Pathology, New York University School of Medicine, NYU Langone Medical CenterNew York, NY, USA; ^2^Department of Radiation Oncology, New York University School of Medicine, NYU Langone Medical CenterNew York, NY, USA

**Keywords:** abscopal effect, fractionation, immunogenic cell death, immunotherapy, inflammation, *in situ* vaccine, radiation regimen, T cells

## Abstract

Ionizing radiation to a cancer site has the ability to convert the irradiated tumor in an immunogenic hub. However, radiation is a complex modifier of the tumor microenvironment and, by itself, is seldom sufficient to induce a therapeutically significant anti-tumor immune response, since it can also activate immune suppressive pathways. While several combinations of local radiation and immunotherapy have been shown in pre-clinical models to induce powerful anti-tumor immunity, the optimal strategy to achieve this effect remains to be defined. When used *in vivo*, radiation effects on tumors depend on the dose per fraction applied, the number of fractions used, and the total dose. Moreover, the interplay of these three variables is contingent upon the tumor setting studied, both in pre-clinical and clinical applications. To enable repair of the collateral damage to the normal tissue, radiation is usually given in multiple fractions, usually of 2 Gy. Generally, the use of larger fractions is limited to stereotactic applications, whereby optimal immobilization reduces inter- and intrafraction movement and permits a very conformal delivery of dose to the target, with optimal exclusion of normal tissue. Translation of the partnership of radiation and immunotherapy to the clinic requires a careful consideration of the radiation regimens used. To date, little is known on whether different dose/fractionation regimens have a specific impact on the anti-tumor immune response. Most experiments combining the two modalities were conducted with single fractions of radiotherapy. However, there is at least some evidence****that when combined with some specific immunotherapy approaches, the ability of radiation to promote anti-tumor immunity is dependent on the dose and fractionation employed. We critically review the available *in vitro* and *in vivo* data on this subject and discuss the potential impact of fractionation on the ability of radiation to synergize with immunotherapy.

## INTRODUCTION

The therapeutic use of local ionizing radiation has been largely guided by a strategy designed to achieve the goal of effectively eliminating cancer cells while causing the least toxicity to normal adjacent tissues. The mechanisms underlying this strategy were defined by Withers as “The 4 R’s of Radiotherapy” (Withers, 1975), an helpful mnemonic reference to the factors thought to determine the response of tissues to fractionated radiation: repair, reassortment, repopulation, and reoxygenation. Steel added a fifth factor, radiosensitivity, in recognition of the fact that the intrinsic vulnerability of different cancer cells differs markedly (Steel et al., 1989).

The choice of fractionating (i.e., delivering the prescribed dose in multiple fractions during separate radiation sessions, usually once/day) derives from the necessity of enabling normal tissue to repair during and after the course of radiation, to exploit the fact that the tumor is at a disadvantage, since its repair machinery is generally damaged. Based on extensive empirical experience, the use of multiple daily doses of about 2 Gy to a total dose of approximately 45–50 Gy, has evolved as a “standard” approach to control microscopic disease for most tumor types, after surgical resection. Generally, higher doses are required when the tumor is in place. However, while total doses as low as 35–45 Gy are sufficient to control most lymphomas, in tumors considered relatively radio-resistant such as melanoma or sarcomas, higher total doses are necessary (Khan et al., 2011).

With the development of image-guided radiotherapy (IGRT) the uncertainty about the target volume is markedly reduced, permitting smaller and more conformal fields that reduce the inclusion of normal tissue and allow the delivery of larger single radiation doses with acceptable complications (Verellen et al., 2007). Particularly when immobilization is assured, and the inter- and intrafraction movement is minimized, single fractions have shown to be both safe and effective. For brain metastases, for example, stereotactic radiosurgery (SRS) utilizes single doses of radiation in the order of 20 Gy, generally achieving control of the lesion for the rest of the patient’s life (Frazier et al., 2010).

These considerations also apply to pre-clinical *in vivo* models. Conversely, studies in cell lines inevitably avoid the issue of normal tissue tolerance, and are generally conducted using a single dose approach. *In vitro*, the radiosensitivity of tumor cells is usually determined by the clonogenic survival assay, which measure the proliferative impairment of cells exposed to various single doses of ionizing radiation, expressed as “surviving fraction”. At least in the first 24 h, there is generally little or no apoptotic response in cancer cells of non-hematopoietic origin (Amundson et al., 2008). In fact, depending on the cell cycle phase at the time of irradiation, carcinoma cells that have lost clonogenic ability have been shown to die by necrosis or by other forms of cell death and after a few or several divisions, up to at least 10 generations post-irradiation (Chu et al., 2002).

Similar to the clinical setting, when radiation is applied in experimental tumors, neoplastic cells, tumor stroma and some adjacent normal tissue are also exposed. *In vivo*, the radiosensitivity of a tumor depends on the complex interaction between the intrinsic sensitivity of the cancer cells and that of the tumor microenvironment, with hypoxia representing a major modulator of radiosensitivity (Vaupel, 2004). Importantly, the results depend on the integrity of the animal immune system. Stone et al. (1979) showed that the radiation dose required to cure 50% of the tumors (TCD_50_) was more than twice as high in mice lacking T cells, providing the first evidence that radiotherapy induces anti-tumor T cell responses that contribute to tumor control.

## RADIATION-INDUCED CELL DEATH, IMPLICATIONS FOR IMMUNOTHERAPY COMBINATIONS

There are at least two important implications of the kinetics of cell death post-radiation. The first is that most irradiated cells survive at least for a limited time, during which they undergo a stress response, transmitted through multiple signal transduction pathways to the surrounding tissue. This process is associated with changes in specific gene expression that depend on the tissue of origin, the genetic background of the host, the p53 status of the tumor and the radiation type and regimen used (Amundson et al., 1999, 2008; Tsai et al., 2007b; John-Aryankalayil et al., 2010). Among genes up-regulated following radiation are those controlling expression of growth factors, cytokines, chemokines, and cell surface receptors that modulate the interaction of the tumor with the immune system (Demaria and Formenti, 2007; Formenti and Demaria, 2008). As an important modifier of the tumor microenvironment, radiation can change tumor immunogenicity with consequences outside of the irradiated field (Formenti and Demaria, 2009).

The second implication is that, after radiation exposure, among the cells programmed to die, the type of death is highly variable, spanning from apoptosis and necrosis to autophagy and mitotic catastrophe. Importantly, radiation has been shown to induce an immunogenic cell death (ICD), characterized by three molecular signals that promote uptake of dying cells by dendritic cells, cross-presentation of the tumor-derived antigens to T cells, and activation of anti-tumor T cells: exposure of calreticulin on the tumor cell surface, release of high-mobility group protein B1 (HMGB1), and release of ATP (Apetoh et al., 2007; Obeid et al., 2007a,b; Ghiringhelli et al., 2009). *In vitro* calreticulin exposure in a mouse colon carcinoma was shown to occur after a single 75 Gy dose (Obeid et al., 2007a), an unrealistic dose to translate to the clinic. However, HMGB-1 release in the EL4 lymphoma occurred after a single 10 Gy dose (Apetoh et al., 2007): currently, little is known about the exact dose-dependency of these effects.

Overall, available evidence suggests that local radiation at clinically therapeutic doses always elicits some activation of the innate and adaptive immune system (McBride et al., 2004). However, the proportion of tumor cells undergoing ICD is variable. Similarly, variable is the type of remodeling of the tumor microenvironment after radiation, for example, in terms of recruiting more functional DC rather than immunosuppressive myeloid cells and regulatory T cells. The results of this balance are likely to determine the ability of radiation to convert the cancer in an effective *in situ* vaccine (Formenti and Demaria, 2012). Understanding this balance has relevant clinical implications, with the potential to expand the application of ionizing radiation.

This review will discuss the available, albeit limited data in support of an effect of dose and fractionation of local radiotherapy in determining successful anti-tumor immunity. We will deliberately exclude discussing the immunosuppression caused by total body radiation, which is largely due to different mechanisms (i.e., deletion of the more sensitive naïve T cells and other cells; McFarland et al., 2012).

## EXPERIMENTS TESTING SINGLE FRACTIONS OF RADIATION

Several studies have shown that ionizing radiation induces or up-regulates cell surface molecules involved in recognition and/or killing of tumor cells by cytolytic T cells (CTL). These include major histocompatibility class I molecules (MHC-I), Fas/CD95, intercellular adhesion molecules-1 (ICAM-1), and NKG2D ligands (Hareyama et al., 1991; Gaugler et al., 1997; Chakraborty et al., 2003, 2004; Kim et al., 2006; Newcomb et al., 2006; Reits et al., 2006). In most studies, escalating doses of radiation, delivered in a single fraction, were tested on one or a few cell lines of mouse or human origin.

In one of the most comprehensive studies, Garnett et al. (2004) analyzed a panel of 23 human tumor cell lines of colon, lung, and prostate origin for the effect of radiation on expression of MHC-I, Fas, ICAM-1, and two tumor-associated antigens, carcinoembryonic antigen (CEA) and mucin-1 (MUC-1). Exposure to a single dose of 10 or 20 Gy induced the up-regulation of at least one molecule in 91% of the cell lines (Garnett et al., 2004). These data indicate that phenotypic changes, which may impact tumor immunogenicity, are common post-radiation. They also highlight the variability among tumors in terms of which molecules are up-regulated. In this respect, the frequent loss or alterations of genes encoding one or more of the molecules required for the generation and assembly of MHC-I in cancer cells, precludes an effective MHC-I up-regulation in response to radiation (Chang and Ferrone, 2007) .

Reits et al. (2006) demonstrated *in vitro* and *in vivo* up-regulation of MHC-I, and provided important insight in the regulation of these molecules by radiation. Human melanoma cells exposed to a single dose of radiation increased MHC-I expression in a dose- and time-dependent manner: while 1 Gy did not cause any significant increase above baseline, 4 Gy slightly increased MHC-I expression. Ten and 25 Gy caused a larger, over twofold, increase at 72 h. A slightly faster kinetic was observed after 25 Gy (Reits et al., 2006). They demonstrated that surface MHC-I expression is enhanced in response to increased availability of antigenic peptides for loading on MHC-I. Within the first 4 h after radiation, an increased degradation of cellular proteins damaged by radiation-induced radicals occurs, and the process evolves to activate mTOR. mTOR activation results in increased protein synthesis and enhances defective ribosomal products. Interestingly, a change in the repertoire of peptides displayed on surface MHC-I molecules was noticeable, with appearance of new peptides not present in non-irradiated tumor cells, reflecting protein synthesis in response to DNA damage (Reits et al., 2006). Employing the mouse MC38 colon carcinoma, Reits et al. (2006) showed that single doses of 8, 10, or 20 Gy enhanced MHC-I expression for up to 11 days: despite the fact that most cells survived irradiation, a larger proportion was eventually eliminated by tumor-specific CTL than those observed in non-irradiated controls. Importantly, *in vivo* there was a synergy between tumor irradiation with a single dose of 10 Gy and adoptive transfer of CTL. The majority of tumors receiving the combination therapy regressed. In contrast, radiation alone only slightly inhibited tumor growth, and CTL transfer by itself had no effect.

Overall, the data suggest that even a radiation regimen relatively ineffective at killing tumor cells and inhibiting tumor growth may still sensitize the tumor to rejection by CTL, if sufficient signaling to repair, triggering mTOR activation, is produced.

Many pro-inflammatory cytokines and chemokines are induced by irradiation of tumors and/or normal tissues. *In vivo*, this is often a reflection of the type of inflammation that develops as an acute or chronic response to the radiation-induced tissue damage (Hong et al., 1999; Johnston et al., 2002; Lugade et al., 2008). *In vitro*, induction of interleukin (IL)-1β was detected as a rapid response to irradiation with a single dose of 20 Gy in normal mouse spleen cells and leukemia cells (Ishihara et al., 1993). IL-1β was also induced in human alveolar macrophages by a single dose of 2 Gy (Degenhardt et al., 2006). IL-1β secretion requires processing by caspase-1, which is activated by the NLRP3 inflammasome (Barker et al., 2011). Death of normal or tumor cells that occurs by apoptosis associated with autophagy is required to activate the inflammasome in macrophages and dendritic cells (Michaud et al., 2011; Petrovski et al., 2011). Activation of the inflammasome and production of IL-1β were identified as essential events for the optimal activation of anti-tumor T cells following treatment-induced ICD (Ghiringhelli et al., 2009). Given that radiation can induce macrophages to release IL-1β in the absence of tumor cells *in vitro* (Degenhardt et al., 2006), it is intriguing to consider whether, *in vivo*, this effect may contribute to the development of anti-tumor immunity after radiotherapy. This could be especially relevant among cancer cells with impaired autophagy pathways that are unable to generate all necessary signals for ICD (Michaud et al., 2011).

Radiation can also directly stimulate the production of some cytokines and chemokines from cancer and/or tumor stromal cells. For example, tumor necrosis factor-alpha (TNF-α) was produced by human sarcoma cells exposed to a single dose of 5 Gy (Hallahan et al., 1989). CXCL16 was induced *in vitro* in human breast cancer cells and murine mammary, prostate, and colon carcinoma cells by a single dose of 12 Gy (Matsumura et al., 2008; Matsumura and Demaria, 2010). Induction of CXCL16 in mouse breast cancer cells was dose-dependent, starting at 2 Gy and reaching a plateau between 6 and 12 Gy. Interestingly, maximal secretion was reached after 6 Gy in one tumor but it required > 12 Gy in another, suggesting inter-tumor variability in the response (Matsumura et al., 2008). Importantly, CXCL16 induction by local radiotherapy with two doses of 12 Gy was also seen *in vivo* in the mouse 4T1 mammary carcinoma, and shown to enhance tumor infiltration by CXCR6^+^ effector CD8 T cells (Matsumura et al., 2008).

Radiation can also induce the activation of anti-inflammatory pathways. For instance, the pleiotropic immunosuppressive cytokine transforming growth factor-beta (TGF-β) was activated by radiation from its latent form after a single dose of 5 and 10 Gy (Barcellos-Hoff, 1993; Barcellos-Hoff et al., 1994; Jobling et al., 2006). TGF-β suppresses the function of dendritic cells and effector CD8 T cells, while promoting the conversion of CD4 T cells into regulatory (Treg) cells (Wrzesinski et al., 2007). Therefore, increased activated TGF-β post-radiation could hinder the development of anti-tumor T cells and their function in the tumor. Strategies to inhibit TGF-β post-radiation are currently investigated in the clinic.

## EXPERIMENTS TESTING DOSE FRACTIONATION

Fewer studies have addressed the effects of dose fractionation. *In vitro*, when mouse B16 melanoma cells were exposed to multiple daily doses of 2 Gy, 5 days/week up to a total dose of 50 Gy, mimicking clinical protocols, MHC-I expression was increased after the second week, when the total dose amounted to 20 Gy (Hauser et al., 1993). The increased expression was stable for at least 5 weeks after the last radiation fraction, and was associated with increased expression of MHC-I heavy chain mRNA, suggesting the possibility that different mechanisms than activation of mTOR (Reits et al., 2006) are responsible for MHC-I up-regulation induced by different radiation regimens.

The contribution of the different mechanisms of MHC-I up-regulation by radiation described *in vitro* remains to be demonstrated *in vivo*. In the B16 murine melanoma model Lugade et al. (2008) found that MHC-I up-regulation after *in vivo* irradiation with a single dose of 15 Gy required host-produced interferon-gamma (IFN-γ) since it was not seen in IFN-γ deficient mice. This suggests that signaling by host cells may dominate *in vivo*, shifting the focus from tumor cell-intrinsic responses to the cross-talk between irradiated tumor cells and the local immunological microenvironment. Consistently, we found that the tumor microenvironment *in vivo* alters the phenotype of cancer cells, as well as their response to radiation. 4T1 mouse breast cancer cells had increased baseline expression of MHC-I *in vivo* as compared to cells cultured *in vitro*, but they lost expression of NKG2D ligands. Radiation increased expression of MHC-I on 4T1 cells *in vitro* but not *in vivo,* while ICAM-1 and Rae-1, one of the NKG2D ligands, were increased by radiation *in vivo* (Ruocco et al., 2012).

Radiation can also up-regulate or induce other molecules that enhance the efficiency of cancer cell killing by CTL. For example, Fas was induced in an *in vivo* mouse model of colon carcinoma after a single dose of 8 Gy or after four fractions of 2 Gy given in consecutive days (Chakraborty et al., 2003, 2004). The two radiation regimens combined with vaccines (vaccinia and avipox recombinant vaccines expressing CEA and three T cell costimulatory molecules) achieved comparable tumor regression, whereas either vaccine or radiation as monotherapy failed to significantly affect tumor growth (Chakraborty et al., 2004). Therefore, Fas expression appears to occur with either single or fractionated RT and results in a clinically detectable effect.

It is tempting to speculate whether this radiation-induced increase in the expression of MHC-I, Fas, or other molecules contributes to better tumor regression particularly in patients with pre-existing higher levels of natural anti-tumor T cells (Galon et al., 2006; Vesely et al., 2011; Wang et al., 2012). In other words, a pre-existing anti-tumor immunity may result to be a predictor for response to radiotherapy.

Using the B16 mouse melanoma, Lee et al. (2009) showed that tumor growth delays obtained with a single dose of 20 Gy or three fractions of 15 Gy were comparable, and almost abrogated by CD8 T cell depletion, suggesting that both regimens can promote cross-priming of anti-tumor T cells. In contrast, 5 Gy × 4 given over a 2-week interval, showed inferior tumor growth inhibition, although the contribution of CD8 T cells to this effect was not investigated. In the same model, Lugade et al. (2005) had previously shown that cross-priming of T cells against tumor antigens was induced in the draining lymph nodes after irradiation with a single dose of 15 Gy or 3 Gy × 5. While both regimens induced the activation and expansion of anti-tumor T cells, and increased VCAM-1 expression on tumor endothelium and T cell infiltration in the tumor, the regimen of 3 Gy × 5 failed to cause a significant inhibition of tumor growth (Lugade et al., 2005).

These results are in contrast with our findings with radiation and anti-CTLA-4. Comparing three radiation regimens, 20 Gy × 1, 8 Gy × 3, and 6 Gy × 5, we demonstrated a marked difference between single dose and fractionated regimens, in the ability to synergize with anti-CTLA-4 antibody treatment and induce an anti-tumor immune response able to inhibit tumor locally, at the irradiated site, and systemically (Dewan et al., 2009). Two poorly immunogenic tumor models not expressing model antigens, a mammary and a colorectal carcinoma, respectively syngeneic to mice of different genetic background were studied. All three regimens had similar ability to cause growth delay of the irradiated tumor, without affecting the growth of a tumor outside of the radiation field. While anti-CTLA-4 by itself or in combination with a single 20 Gy dose was ineffective, when combined with the two fractionated regimens it significantly improved inhibition of both the irradiated and tumors outside the irradiated field (abscopal response, from *ab-scopus*, i.e., outside the target). The effectiveness of the generated anti-tumor response was highest with 8 Gy × 3, with 80% of the irradiated tumors and 40% of the tumors outside the field regressing completely. Since anti-CTLA-4 antibody is known to be ineffective against poorly immunogenic tumors but to synergize with vaccination in inducing anti-tumor immunity (Peggs et al., 2008), these data imply that radiation used as single dose of 20 Gy failed to convert the tumor into an *in situ* vaccine. These results suggest that, for the combination with anti-CTLA-4, there may be an optimal window for the pro-immunogenic effects of radiation, with a hypofractionated regimen providing the best results. We are currently performing genome-wide gene expression analyses to investigate the changes that distinguish the two regimens and may be responsible for the interaction of the irradiated tumor with the immune system.

Overall, while some degree of immunization against the tumor may be always promoted by radiotherapy, the magnitude of this effect and the overall changes in the tumor toward a more or less immunosuppressive environment are likely to be the determinants of treatment success. Pre-clinically, a specific dose and fractionation may be superior to another, and it appears to be model-dependent. In this regard, Schaue et al. (2012) recently proposed that the relative ability of a given radiation regimen to increase cross-priming while not increasing Treg cell numbers will determine its pro-immunogenic effect. They identified a hypofractionated regimen with two fractions of 7.5 Gy as providing the best compromise between promotion of T cell cross-priming to tumor antigens versus relative induction of Treg (measured as increased Treg cell numbers) in the mouse B16 melanoma model. The mechanisms underlying Treg cells increase by radiation as well as their suppressive function in this setting remain to be clarified (Qu et al., 2010; Billiard et al., 2011; Kachikwu et al., 2011).

## LEARNING FROM CLINICAL EXPERIENCE

Local radiation by itself may occasionally be able to elicit the development of a sustained anti-tumor immune response able to control tumor locally and systemically as suggested from reported cases of abscopal effects, i.e., tumor responses outside of the field of radiation in cancer patients receiving radiation to one site (Mole, 1953; Ehlers and Fridman, 1973; Rees and Ross, 1983; Ohba et al., 1998; Wersall et al., 2006). In one report, 4 of 28 patients with metastatic renal cell carcinoma showed abscopal effects: in two patients after receiving 8 Gy × 4 to the primary tumor by SBRT, and in another two patients after two doses of 15 Gy to a metastatic site (Wersall et al., 2006). The lack of data about the immune response in these patients makes it difficult to reach any definite conclusion about the involvement of the immune system.

However, immunological changes associated with an abscopal response were recently reported in a melanoma patient treated with local radiotherapy and ipilimumab, the anti-CTLA-4 antibody approved for clinical use (Postow et al., 2012). The patient received radiotherapy in three fractions of 9.5 Gy, a regimen comparable to the regimen (8 Gy × 3) showing optimal synergy with anti-CTLA-4 therapy in mouse tumor models (Dewan et al., 2009). The patient displayed a complete clinical remission of the primary and metastatic sites, despite previous progression with ipilimumab therapy when given alone.

Abscopal responses were also reported in a clinical study of patients with low-grade B cell lymphoma treated with 2 Gy × 2 to a single tumor site that was injected with a Toll-like receptor 9 (TLR9) agonist PF-3512676, an activator of B cells and antigen-presenting cells (Brody et al., 2010).

It has been speculated that conventional fractionated radiotherapy with multiple fractions of about 2 Gy is immunosuppressive (Lee et al., 2009). However, clinical data disproves this contention: Gulley et al. (2005) administered a poxviral vaccine encoding prostate-specific antigen (PSA) to 17 prostate cancer patients undergoing radiotherapy with total external beam dose ≥70 Gy given in 1.8 to 2.0 Gy per fraction. PSA-specific T cell responses were analyzed before radiotherapy, immediately after and 3 months later. Eight patients showed blunted immune responses to PSA following radiotherapy, six had stable responses, and in two patients the response was increased after radiotherapy (Gulley et al., 2005). Moreover, six out of eight patients evaluated showed the development of T cell responses against tumor antigens not present in the vaccine, suggesting that radiation promoted the activation of T cells against other tumor antigens (Gulley et al., 2005).

Finally, a recent report in patients with metastatic melanoma and renal cell carcinoma treated with SBRT given in one, two, or three doses of 20 Gy, in combination with high dose IL-2 showed a response rate in non-irradiated lesions (abscopal responses) higher than expected with IL-2 alone (Seung et al., 2012).

Overall, while emerging clinical data confirm at least some of the observations in experimental models, they fail to provide indications about the best radiation regimen to be used to elicit anti-tumor immune responses. One limitation in interpreting these results is the lack of randomized studies comparing radiation regimens for their ability to synergize with immunotherapy.

## CHALLENGES AND FUTURE DIRECTIONS

While data about the effects of radiation on various immune parameters is rapidly accumulating in experimental model, potential pitfalls exist in correctly interpreting the results. For instance, tumors which are more immunogenic, for example, engineered to express a “model” antigen such as ovalbumin (OVA), tend be more susceptible to immune-mediated rejection than poorly immunogenic tumors. The latter, however, much better mimic the reality of clinical cancer patients, who generally are diagnosed after immune-selection had edited out more immunogenic antigens (Vesely et al., 2011).

Tumor size and intrinsic radiosensitivity also affect the degree of response to a tested intervention. Importantly, pre-clinical evidence of an induced immune response based on re-challenging with the same experimental tumor initially used to immunize remains only an indirect predictor for a successful therapeutic paradigm. In fact, in the clinic the endpoint is control of pre-existing micro- or macrometastatic foci and impact on survival, a much harder goal to achieve. Additionally, different immunosuppressive mechanisms (e.g., Treg cells versus myeloid-derived suppressor cells) may dominate the models compared. This variability is likely to also exist in different clinical tumor types.

For example, there is some evidence that radiation promotes the immunosuppressive function of macrophages (Tsai et al., 2007a). On the other hand, a recent report shows that radiotherapy induces the production of type I IFN by myeloid cells infiltrating the tumor and promotes the development of anti-tumor immunity (Burnette et al., 2011). Whether these contrasting effects are dependent on the radiation regimen or the tumor model employed remains to be established.

Therefore, conclusions about the relative efficacy of different radiation regimens can only be made by direct comparison using the same, unmodified tumor model and testing the combination with the same immunotherapy strategy.

Finally, since we are witnessing a paradigm change in the use of radiotherapy that promises to revolutionize patient treatment (Formenti and Demaria, 2012), the development of a common language is essential. A cautious choice of terminology is warranted. For instance, terms as “ablative” should be reserved to settings where a complete elimination of tumors is achieved. In addition, attributes like “conventional” and “standard” need to be carefully justified. This is especially needed since radiation biology is becoming a point of encounter for other specialties.

## CONCLUSIONS

Tumor rejection by the immune system involves a common final pathway mediated by the activation of a specific set of genes (Wang et al., 2008). These include the coordinate activation of IFN-stimulated genes and immune effector functions. However, multiple factors serve as checkpoints in the pathway toward this canonical response (Wang et al., 2012). In order to identify the radiation regimens that can best overcome the checkpoints toward immune-mediated tumor rejection, it should be possible to identify a gene signature that defines the pro-immunogenic effects of radiation. Such signature is the result of the interaction between the pre-existing tumor microenvironment, the genetic predisposition of the host, and the radiation regimen used.

In support of this concept, Tsai et al. (2007b) reported distinct molecular responses of human breast, prostate, and glioma tumor cells exposed to single dose (10 Gy) versus fractionated (2 Gy × 5) radiation *in vitro* and *in vivo*. Importantly, selective up-regulation of IFN-related genes by fractionated but not single dose radiation was seen in all three cell lines (Tsai et al., 2007a). In addition, a comparison of the response of the prostate carcinoma cell line to single dose and fractionated radiation *in vitro* versus *in vivo* showed that there was no overlap between the four conditions, indicating that the gene response to radiation is highly dependent on the microenvironment and the regimen (Tsai et al., 2007a). Although in this study the recipient mice were T cell deficient, they had an intact innate immunity, which is likely to play a key role in the initial triage of tissue damage. John-Aryankalayil et al. (2010) showed in human prostate cancer cells that genes regulating immune and stress response, cell cycle, and apoptosis were significantly up-regulated by multi-fractionated radiation compared to single-dose radiation.

Although the optimal pro-immunogenic radiation regimen may not necessarily be the same for all tumor types or settings, a signature that defines the pro-immunogenic effects of radiation could be used to optimize protocols of radiation and immunotherapy in different tumor types, as well as to predict response to treatment in different patients.

## Conflict of Interest Statement

The authors declare that the research was conducted in the absence of any commercial or financial relationships that could be construed as a potential conflict of interest.
